# Transitioning from microscopy to PCR for protozoa in Norway – Impact on detection of protozoa and helminths: A register study

**DOI:** 10.1017/S0950268825100228

**Published:** 2025-06-26

**Authors:** Lars Sandven, Hanne Brekke, Tore Lier, Liv Reidun Tverelv, Jan Egil Afset, Audun Sivertsen, Kurt Hanevik

**Affiliations:** 1 Department of Internal Medicine, Førde Central hospital, Førde, Norway; 2 Department of Infectious Diseases, Haukeland University Hospital, Bergen, Norway; 3 Department of Medical Microbiology, Oslo University Hospital Ullevål, Oslo, Norway; 4 Department of Microbiology and Infections Control, University Hospital of Northern Norway, Tromsø, Norway; 5 Department of Clinical and Molecular Medicine, Faculty of Medicine and Health Sciences, Norwegian University of Science and Technology, Trondheim, Norway; 6 Department of Medical Microbiology, St. Olavs Hospital, Trondheim University Hospital, Trondheim, Norway; 7 Department of Microbiology, Haukeland University Hospital, Bergen, Norway; 8 Department of Clinical Science, UiB, Bergen, Norway; 9 National Centre for Tropical Infectious Diseases, Haukeland University Hospital, Bergen, Norway

**Keywords:** *cryptosporidium*, enteropathogenic, Europe, *Giardia lamblia*, immigrant screening, microscopy, PCR

## Abstract

The aim of this study was to describe how the detection of protozoan and helminth parasites has been affected by the introduction of polymerase chain reaction (PCR) and changes in test algorithms. We extracted data about faecal samples tested for parasites (n = 114839) at five Norwegian clinical microbiology laboratories. Samples were classified into prePCR or postPCR depending on whether they were submitted before or after the introduction of PCR, and into diagnostic episodes (n = 99320). The number of diagnostic episodes increased 3.7-fold from prePCR to postPCR. Giardia positive episodes doubled, the positivity rate decreased from 2.0% to 1.3%. Cryptosporidium was hardly detected prePCR and increased to a positivity rate of 1.2%. Entamoeba histolytica was rarely found. Episodes examined for helminths decreased 51%, the number of positive episodes decreased 34%. Samples from immigrants were more likely to be positive for Giardia, E. histolytica, or helminths and less likely to be Cryptosporidium positive. During the COVID-19 pandemic, the number of Giardia and helminth-positive episodes decreased. Cryptosporidium-positive episodes remained unchanged. The implementation of multiplex PCR for protozoa led to a doubling of Giardia cases and a better test for Cryptosporidium. Fewer microscopy examinations raise concerns that helminth infections may be overlooked.

## Introduction

Intestinal parasites cause morbidity and mortality worldwide. In high-income countries, their prevalence is low. They are often considered only in returning travellers and immigrants, although for *Giardia*, this is being challenged by new European data [1]. Detection has traditionally been by microscopy, and three faecal samples are normally requested and examined to assure sufficient sensitivity [2]. Some parasites, like *Cryptosporidium* and *Cyclospora*, cannot be reliably detected without specific staining procedures, which are usually performed only when clinical suspicion is high. Lack of awareness about these parasites limits such requests [3].

The protozoan parasites *Giardia* and *Cryptosporidium* are the most common intestinal protozoa causing gastroenteritis in both high- and low-income countries. The most common clinical presentation is protracted diarrhoea of moderate severity, but infections range from being asymptomatic to causing severe dehydration and malabsorption [2]. Both of these protozoa may cause chronic infection, and *Cryptosporidium* can be a serious problem in immunocompromised patients [4]. In the Norwegian population of around 5 million people, about 400 cases of cryptosporidiosis and 500 cases of giardiasis have been reported annually to the Norwegian registry for notifiable infectious diseases [5] during the last few years. Cyst and oocyst forms of the parasites are chlorination-resistant and easily transmitted in water. In Scandinavia, there have been large waterborne outbreaks of giardiasis [6] and cryptosporidiosis [7, 8]. Both infections can cause long-term sequelae, including post-infectious irritable bowel syndrome and fatigue [9, 10].

*E. histolytica* is an uncommon cause of parasitic infections in Europe [11]. Microscopy examinations cannot distinguish between pathogenic *E. histolytica* and non-pathogenic *Entamoeba dispar* [11]. *E. histolytica* is often included in PCR panels because of its potential for severe infections.

In recent years, many high-income countries have introduced PCR panels as first-line screening for protozoan parasites in faeces. European clinical laboratories usually include the protozoa *G. lamblia*, *Cryptosporidium*, and *E. histolytica* in multiplex PCR panels for diarrhoeal disease. These panels also detect viruses, bacteria, and specific virulence genes. Along with the introduction of new diagnostic methods, the algorithms for faecal sample testing were also adapted. Before the introduction of PCR, only faecal samples from patients considered high risk were examined for parasites. These were usually samples from travellers or immigrants. After the introduction of PCR, most faecal samples were examined with a multiplex PCR examining for protozoa, along with PCR panels for other viral and bacterial common pathogens causing diarrhoea.

There is an extensive body of studies addressing the diagnostic accuracy of the transition from microscopy to PCR for intestinal protozoa [12–15].

There is limited data on how the introduction of PCR has changed the magnitude and demography of the sampled population and the positivity rates. It is not known if the introduction of PCR has caused a decrease in faecal samples being examined for parasites not included in the protozoa PCR panel.

The aim of this study was to assess the impact of introducing PCR and an altered testing algorithm for the detection of intestinal protozoan parasites and helminths in Norway.

## Methods

We conducted a multicentre, retrospective registry study with participating centres from all healthcare regions in Norway. Four tertiary centres participated: Haukeland University Hospital (HUS), Bergen and district hospital in Førde (receiving samples from almost all general practitioners (GPs) and hospitals in Vestland county); the University Hospital of North Norway, Tromsø (receiving most samples from GPs and hospitals in the counties Troms, Finnmark); Oslo University Hospital (OUS), Oslo (receiving samples from hospitalized patients at the major hospital in Oslo); and St. Olavs Hospital (STO), Trondheim (receiving most samples from GPs and hospitals in Trøndelag county).

Data were extracted from electronic patient registries at participating clinical microbiology departments for all faecal samples examined for ova and cysts by microscopy, Enzyme-linked immunosorbent assay for *E. histolytica* or PCR for parasites, at participating centres between 2014 and 2021. Samples were generally from patients presenting with gastrointestinal symptoms. After the introduction of PCR, most laboratories examined all samples for protozoa, with Oslo and Trondheim continuing to be a bit selective regarding what samples were subject to examination for protozoa.

Faecal samples to be examined with microscopy were prepared using the formalin/ether method and then centrifuged. The precipitate was examined by light microscope with iodine staining. Samples with a high clinical suspicion of *Cryptosporidium* were stained with the modified Ziehl–Neelsen method. Three samples were recommended when examining for parasites with microscopy.

Samples to be examined with PCR were subject to DNA extraction using automated kit solutions (Supplementary Table S1) before being used in kit-based multiplex PCR reaction mixes and run on a real-time PCR thermocycler. One sample was recommended for examinations with PCR.

Participating centres had different strategies to separate *E. histolytica* from *E. dispar* before the introduction of PCR. One of these was that microscopy findings of *E. histolytica/dispar* led to a request for a new, fresh sample for ELISA testing. This was done outside of normal routines, and quite often, a second sample was not received. We only received reliable data about the ELISA testing from Oslo University Hospital.

Sample registration date, patient age cohort, sex, and test results were collected locally from electronic patient registries at the laboratories. Age was grouped into 10-year cohorts.

Samples were assigned to the prePCR period if they were registered earlier than the date for introducing PCR at the respective centre. If not, the sample was assigned to the postPCR period. For calculations and graphs at the episode level, the first date in the episode was used. An episode spanning the date of introducing PCR was assigned to the prePCR group.

Participating centres received samples from practising specialists, hospitals, and from the primary health care (general practitioners and nursing homes). In Bergen, Oslo, and Trondheim, specialized units working only with immigrants’ health ordered samples, enabling specific analyses of immigrants as a group. Denominator data for population per age group were collected from Statistics Norway.

When a patient submitted more than one sample within a 60-day period, the samples were analysed as part of the same episode. Most patients with giardiasis, and also cryptosporidiosis, will respond to treatment or eradicate the parasite spontaneously within 5 weeks [16]. Unsuccessfully treated patients could be assumed to return for further evaluation, and possibly new samples, within 60 days. Therefore, all subsequent samples were considered part of the same episode until there was a 60-day period without a new sample being submitted. The risk of reinfection within this period was considered negligible.

The COVID-19 pandemic affected infectious disease prevalence, including transmission of enteric parasitic diseases. The Norwegian government introduced COVID-19 restrictions on 12 March 2020.

Data were collected and analysed using Excel (Microsoft, One Microsoft Way, Redmond, Washington, USA). All centres had different durations of the period of observation before and after PCR was introduced. Absolute numbers are therefore presented as numbers per observation year.

As a retrospective registry study with a large number of participants, this study fulfilled the criteria for not obtaining written consent from patients. The study was approved by the Regional Committee for Medical and Health Research Ethics South-Eastern Norway (no. 399585). The data protection officer at each participating centre also approved the study.

## Result

We extracted registered data for 129614 faecal samples submitted to the five participating centres between 2014 and 2021. Samples only examined for bacterial virulence genes or enteropathogenic viruses and not for parasites were excluded (*n* = 11398). Thus, 114839 samples submitted for ova and parasites microscopy (F-micro) and/or PCR for faecal pathogens (F-PCR) remained. Of these, 17030 were from the period before the introduction of PCR (prePCR) and 97809 samples after the introduction (postPCR). Samples examined by both PCR and microscopy were counted as one sample (Table 1). PCR methods were introduced at different time points at each of the participating centres (Table 1). The number of examined samples increased 3.4-fold from a mean of 5929 per year in the prePCR to a mean of 20402 per year in the postPCR period. The number of samples analysed by microscopy decreased by 34% from 5922 to 3934 per year.

**Table 1.** Date of PCR introduction, years of observation, and samples included per centre from 2014 to 2021

		prePCR period		postPCR period				
Centre	Faeces PCR introduced	Observation time (years)	F-micro Total	Observation time (years)	F-micro	F-PCR	F-PCR & F-micro	Total
FSS	03.12.2019	5.92	1793	2.08	46	2028	29	2103
HUS	19.04.2017	3.30	7704	4.70	2058	37952	3325	43335
OUS	12.01.2015	1.03	1637	6.97	1586	11612	3949	17147
STO	01.12.2015	1.92	2062	6.09	465	26729	2830	30024
UNN	19.02.2020	6.14	3834	1.87	199	4732	269	5200
Total			*17030*		4354	83061	10402	*97809*

FSS = Førde Central Hospital, HUS = Haukeland University Hospital, OUS = Oslo University Hospital, STO = St. Olavs hospital Trondheim University Hospital, UNN = University Hospital of North Norway.

Samples submitted by the same patient within a 60-day period were grouped into diagnostic episodes (*n* = 99320). The proportion of diagnostic episodes originating from specialized health care increased from 30% of all episodes prePCR (mean 1423 episodes/year), to 41% of all postPCR episodes (mean 7238 episodes/year), while the proportion of episodes from GPs decreased accordingly.

### Number of samples per episode

When analysing episodes examined by microscopy, the proportion of episodes where three or more samples were examined increased from 4.6% (*n* = 662) prePCR to 7.7% (*n* = 958) postPCR (Supplementary Table S2). There was an increase in episodes containing two samples from 6.3% (*n* = 1579) in the prePCR to 15.0% (*n* = 1873) in the postPCR period.

### Demographics

**Figure 1.**
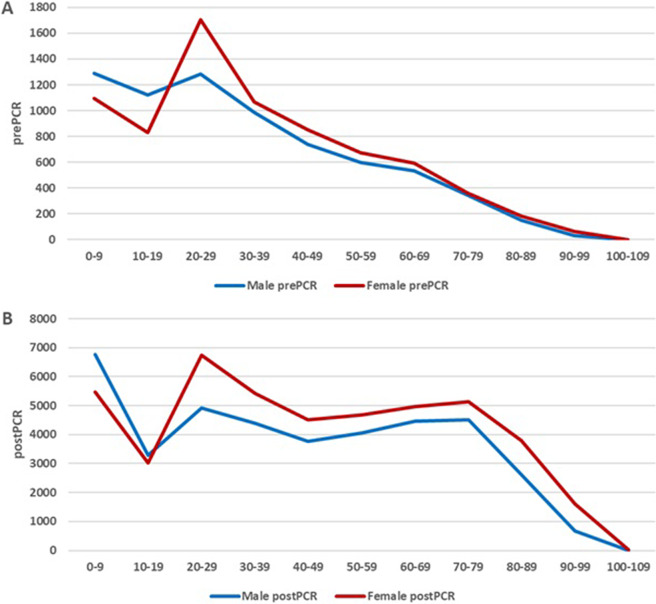
Diagnostic episodes by age. A prePCR. B postPCR.

Before and after PCR, more of the examined episodes were from women (*n* = 52787) than from men (*n* = 46529). The overall gender difference increased slightly after the introduction of the PCR, with the proportion of females rising from 51.2% to 53.5%. In both prePCR and postPCR periods, there was a preponderance of diagnostic episodes in male children, while in adults, there was a preponderance of females being examined for parasites (Figure 1). Out of the episodes positive for any parasite, men represented a higher proportion than women in both the prePCR (57.7%) and postPCR (52.3%) periods.

In the prePCR period, patients above 50 years of age were less frequently examined for parasites. PostPCR, this trend changed with testing for protozoa being performed also in the older age groups (Figure 1). When correcting for the naturally decreasing number of people in the older age groups, this trend was more profound (Supplementary Figure S1).

### Impact on protozoa detection

**Figure 2.**
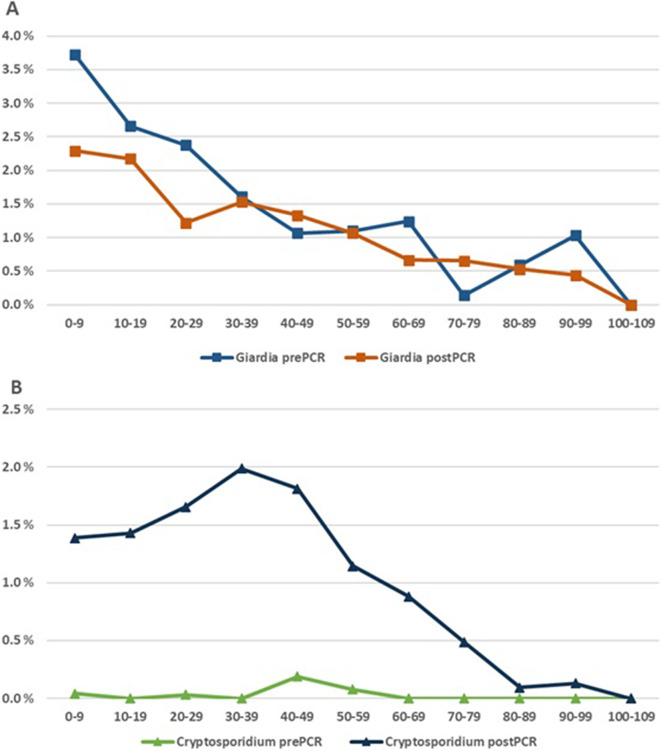
Positive ratio of episodes by age. A. *Giardia* prePCR and postPCR. B. *Cryptosporidium* prePCR and postPCR.

In the prePCR period, 4829 episodes/year were examined for *Giardia*, increasing to 17704 episodes/year (3.7-fold increase) after the introduction of PCR for intestinal protozoa. During the prePCR period, *Giardia* was detected in an average of 109 episodes/year. This number rose twofold to 218 episodes/year with PCR (Table 2). However, the mean positivity rate for *Giardia* decreased from 2.0% to 1.3%. This decrease was especially marked in children (Supplementary Figure S2) (Figure 2).

**Table 2.** Average positive diagnostic episodes per year per centre (ratio of positives by examined episodes)

	Giardia lamblia		Cryptosporidium		Helminths	
Centre	prePCR	postPCR	prePCR	postPCR	prePCR	postPCR
FSS	1.7 (0.6%)	10.1 (1.1%)	0.0 (0.0%)	25.5 (2.9%)	1.2 (0.4%)	1.0 (3.2%)
HUS	41.2 (2.0%)	103.7 (1.3%)	0.3 (0.0%)	84.8 (1.1%)	48.2 (2.3%)	22.5 (2.1%)
OUS	23.3 (2.3%)	26.5 (1.3%)	4.9 (0.5%)	16.8 (0.8%)	4.9 (0.5%)	8.9 (1.5%)
STO	33.4 (3.7%)	57.8 (1.3%)	0.0 (0.0%)	74.7 (1.7%)	11.0 (1.2%)	14.0 (3.0%)
UNN	9.8 (1.9%)	19.8 (0.8%)	0.0 (0.0%)	7.0 (0.3%)	8.0 (1.6%)	1.6 (0.8%)
*Sum*	*109 (2.0%)*	*218 (1.3%)*	*5 (0.04%)*	*209 (1.2%)*	*73.2 (1.7%)*	*48.0 (2.1%)*

FSS = Førde Central Hospital, HUS = Haukeland University Hospital, OUS = Oslo University Hospital, STO = St. Olavs Hospital Trondheim University Hospital, UNN = University Hospital of North Norway).

For *Cryptosporidium*, the introduction of PCR meant moving from a situation where this pathogen was very rarely looked for to being routinely examined for. During the prePCR period, *Cryptosporidium* was found in only about five episodes/year. This increased to 209 episodes/year in the postPCR period. After PCR was introduced, the number of *Cryptosporidium* positive episodes at each centre was quite stable (Supplementary Table S3). Data regarding the number of samples that were stained specifically for microscopy of *Cryptosporidium* were not available.

The dataset contained a few episodes with suspected or verified *E. histolytica* infections. The number of episodes with suspected or positive *E. histolytica* decreased by 72%, from 51.5 episodes/year prePCR to 14.5 episodes/year postPCR. Based on the collected data, it became evident that there were different practices to differentiate *E. dispar* from *E. histolytica* in the prePCR period. Sometimes a microscopy result showing potential *E. histolytica* was not confirmed by ELISA or PCR. Numbers from the prePCR period are therefore uncertain regarding *E. histolytica.*

### Impact on enteropathogenic helminth detection

The number of episodes examined by light microscopy decreased by 51% after the introduction of PCR for enteropathogenic protozoa, from 4823 episodes/year to 2366 episodes/year (Supplementary Table S4).

In the prePCR period, enteropathogenic helminths were detected in an average of 73.2 episodes/year. This decreased to 48.0 episodes/year after PCR for pathogenic protozoa was introduced, a 34% reduction (Table 2).

### Impact of COVID-19 restrictions

Travel restrictions were implemented in Norway on the 12 April 2020. To evaluate the impact of COVID-19 restrictions, we compared data from the 17 months before travel restrictions to the 17 months with restrictions (Supplementary Table S5). The number of examined diagnostic episodes decreased in the travel restriction period from a mean of 18286 to 15025 per year.

With travel restrictions, the detection rate for *Giardia* fell from 1.0% to 0.6% of submitted samples. The detection rate for *Cryptosporidium* increased slightly from 1.1% before travel restrictions to 1.3% with restrictions. Approximately the same number of *Cryptosporidium*-positive episodes were being diagnosed in these two periods (Table 3). The number of helminths detected per year decreased from 50 to 35 in the travel restriction period. The positivity rate increased from 1.7% to 2.3%.

**Table 3.** Annual diagnostic episodes during the period 17 months before and 17 months after the introduction of COVID-19 restrictions on 12 March 2020. Positivity rate, percentage, in brackets

	Pre Covid-19 restrictions	With Covid-19 restrictions
Total episodes	18286	15025
Episodes examined for helminths	2961	1522
All parasite-positive episodes	521 (2.9)	356 (2.4)
*Giardia lamblia*	260 (1.4)	127 (0.8)
*Cryptosporidium*	192 (1.0)	189 (1.3)
*Entamoeba histolytica*	19 (0.1)	4 (0.0)
Helminths	50 (1.7)	35 (2.3)

FSS = Førde Central Hospital, HUS = Haukeland University Hospital, OUS = Oslo University Hospital, STO = Trondheim University Hospital, UNN=University Hospital of North Norway.

### Immigrants

Data regarding samples submitted from immigrant health services were available from three centres (HUS, STO, OUS). Data were analysed only for the postPCR period. We compared the episodes originating from immigrant health units to episodes not ordered by such units.

Episodes from immigrants were 7.6 times as likely to be positive for *Giardia* compared to episodes not from immigrants (Table 4). The same trend was seen for *E. histolytica* and helminths. *Cryptosporidium* was rarely found in samples from immigrants (Table 4).

**Table 4.** Episodes examined for protozoa by PCR and/or microscopy and for helminths by microscopy at centres receiving samples from an immigrant’s health unit (HUS, STO, OUS). Positivity rate, percentage, in brackets

	Non- immigrant		Immigrant	
	Tested	Positive	Tested	Positive
*Giardia*	77220	916 (1.2)	1217	109 (9.0)
*Cryptosporidium*	77220	969 (1.3)	1217	2 (0.2)
*Entamoeba histolytica*	77220	63 (0.1)	1217	20 (1.6)
Helminths	10934	202 (1.9)	1069	51 (4.8)

## Discussion

We examined a large dataset of test results from faecal samples examined for parasites at five Norwegian clinical microbiological laboratories over 8 years. During this period, multiplex quantitative PCR (qPCR) panels were introduced, and sample-processing algorithms were adapted. This resulted in a large expansion in the number of samples analysed for the protozoa *Giardia, Cryptosporidium*, and *E. histolytica.* It led to the establishment of a good diagnostic option for *Cryptosporidium* that hardly existed before, an increase in *Giardia* positive samples, and a reduction in *E. histolytica* detections. However, it is also evident that laboratories shifted to do fewer microscopy examinations for eggs and cysts, causing a considerable reduction in detected helminth parasites.

### Episodes, gender and age

Converting samples into diagnostic episodes allowed correction for multiple samples from the same person and comparison across the periods.

The double-peaked curve for both diagnostic episodes and protozoan parasite positivity by age is well known. However, the peak in adults seems to come earlier in our data than in similar data from the United States [17]. It has been shown that this bimodal pattern has gradually become less pronounced over the last decades in the United States [17, 18].

In the postPCR period, a higher proportion of the examined episodes originated from specialized health care centres, in which patients are normally older. Interestingly, the positivity rate for *Giardia* in middle and old age was largely unaltered from prePCR to postPCR period, revealing that these parasites can indeed be present at all ages.

More women were tested for parasites, while more men were diagnosed with intestinal parasites in our results. This could be due to differences in health-seeking behaviours or gender biased gastrointestinal complaints such as irritable bowel syndrome. The higher protozoa positivity rate in men could be an effect of higher occupational exposure (farming, plumbing, sewage reconstruction, etc) or recreational activities such as hunting or hiking.

In the microscopy era, three samples were recommended to give a sensitivity of around 90% for giardiasis. Due to the high sensitivity of PCR, one faecal sample is enough to give equal, or even better, sensitivity [19, 20]. Only 4.6% of the episodes prePCR were examined with the recommended number of three or more samples. This raises concern that the rather low sensitivity of microscopy for parasites found in many studies [21] may in fact have been even lower in clinical practice.

### Diagnostic options and accuracy

The shift to PCR-based diagnostics led to changed testing algorithms and a more uniform approach. After the introduction of PCR, most samples were examined with multiplex PCR assays targeting common viral, bacterial, and parasitic pathogens. With PCR, the number of episodes examined per year for *Giardia* increased 3.4-fold, and the number of *Giardia-*positive episodes per year doubled. This reflects the inclusive testing algorithms in the PCR period, where clinical suspicion of *Giardia* infection was no longer necessary to be tested for this pathogen, and the increased sensitivity led to the detection of *Giardia* in patients that would not have been detected prior to PCR.

We observed a dramatic increase in episodes where *Cryptosporidium* was diagnosed. Tedious staining methods, low sensitivity, and a lack of awareness about cryptosporidiosis among Norwegian clinicians probably contributed to low detection frequency prior to the introduction of PCR. The self-limiting nature of the infection in immunocompetent patients may also have discouraged frequent examination for this pathogen. Still, diagnosing *Cryptosporidium* infections is important for mapping transmission and detection of outbreaks. The parasite has been responsible for huge waterborne outbreaks in the United States and in Sweden [7, 22]. A recent study identified two potential small outbreaks of *Cryptosporidium* in Norway [23]. Confirming a diagnosis that explains the symptoms can reassure patients and prevent further unnecessary medical examinations. The increase in cryptosporidiosis cases seen in Norway over the last decade is largely due to a gradual increase in the number of clinical laboratories introducing PCR for protozoa. When PCR was introduced, there was a stable number of *Cryptosporidium* episodes detected (Supplementary Table S3).

Rapid and accurate diagnosis of parasitic infections is a cornerstone of reducing the impact of waterborne outbreaks. Clinically, gastrointestinal infections caused by parasites are difficult to distinguish from gastroenteritis from other pathogens [3]. Selective analysis of protozoa, mostly in patients with a history of travel abroad, contributed to the delayed detection of the *Giardia* outbreak in Bergen in 2004 [6]. Screening patients with low clinical suspicion could therefore lead to faster outbreak detection and control.

When PCR became available, fewer episodes were examined for helminths and fewer helminths were diagnosed. It is a concern that parasites not included in PCR panels could be increasingly overlooked. The decrease in helminth-positive episodes is not as profound as the decrease in the number of episodes tested. This indicates that episodes with a higher clinical suspicion are still examined by microscopy. The climate and hygienic standards in Europe do not support the transmission of most enteropathogenic helminths. The argument for detecting outbreaks is therefore less important for helminths than for waterborne protozoa. In addition, serology and eosinophilia are often better clinical tools to evaluate and diagnose potentially serious helminth infections like schistosomiasis and strongyloidiasis. Accurate expanded nucleic-acid-based test panels for a broader set of intestinal parasites are now being introduced in many clinical laboratories. These tests might allow detection rates for at least some helminths to bounce back. Still, clinicians need to be aware of which pathogens are not included in the PCR panel they utilize, especially for clinically important parasites like *Strongyloides* and *Schistosoma* spp.

### Impact of pandemic restrictions

During the pandemic, there were fewer episodes tested for intestinal protozoa. Probable explanations for this could be that fewer patients were infected with communicable diarrhoeal diseases. It is known that social distancing resulted in fewer infections with communicable diseases like viral gastroenteritis [24].

The decrease seen in *Giardia* positive episodes is probably due to the arrival of fewer immigrants and less travelling abroad. The decrease in the *Giardia* positivity rate indicates that more of the episodes tested were caused by other pathogens.

Interestingly, this pandemic-related decrease in incidence was not seen for *Cryptosporidium.* A plausible interpretation is that *Cryptosporidium* is an autochthonous infection in Norway, with transmission cycles largely unbroken by the pandemic. Possibly more patients were infected during the pandemic because of more leisure time spent at cottages in rural Norway. Trips to cottages often involve drinking untreated water from wells or streams and closer contact with animals.

During the pandemic, the diagnostic positivity rate for helminths increased while the number of positive episodes decreased. This could mean that episodes with high suspicion of helminth infections were still examined during the pandemic.

### Immigrant screening

The lower positivity rate of *Cryptosporidium* in immigrants compared to the general population was unexpected. However, the majority of samples from immigrants are part of a screening program in non-symptomatic individuals. As *Cryptosporidium* is less prone to prolonged shedding and asymptomatic carriage than *Giardia* [25, 26], the identified difference makes sense.

For *Giardia, E. histolytica*, and helminths, the positivity rate was higher in the immigrant population. Immigrants in Norway often come from areas endemic for these parasites. It reflects the very prolonged nature of infections these pathogens may have in some individuals. The findings support the usefulness of screening immigrants using microscopy as well as PCR. A positive side effect is that immigrant screening programs help microscopists practice their skills.

### Strengths and limitations

The present study is retrospective and utilizes data from hospital laboratory information systems. Data were extracted by each centre before being integrated into one dataset. Our data may therefore be biased by differences in registration and extraction at different sites. To reduce the effect of such differences, a standardized registration layout was used by all participating laboratories. Using registry data, however, assures that our data are a representation of real clinical practice.

In Norway, all inhabitants have a unique social security number that is used to identify patients in electronic patient registries. This is a strength, ensuring that repeated samples from the same patient could be accurately aggregated into diagnostic episodes.

The pandemic travel restrictions affected our data. We have tried to account for this by repeating our calculations with all samples taken in the pandemic period excluded. We found the same trends in this sub-analysis but acknowledge that the restrictions add some uncertainties to our analyses.

## Conclusion

The transition from microscopy to PCR for intestinal parasites led to a large increase in the number of episodes examined for three important protozoan parasites with more sensitive methodology. There was a shift from only travel-related, or prolonged diarrhoea being tested to all diarrhoea cases being examined for parasites. Consequently, more episodes with *Giardia* and *Cryptosporidium* were detected. This increase included the older age groups, who had rarely been examined for parasites before PCR was introduced. Fewer episodes were examined for helminths, and fewer helminth-positive episodes were detected. Screened immigrants were less likely to be *Cryptosporidium* positive and more likely to harbour *Giardia, E. histolytica*, or helminths. The detection rate of *Giardia, E. histolytica*, and helminths considerably decreased during the Covid-19 pandemic restrictions, while *Cryptosporidium* incidence was not affected. Our findings support that *Cryptosporidium* is endemic in Norway.

## Supporting information

10.1017/S0950268825100228.sm001 Sandven et al. supplementary material Sandven et al. supplementary material

## Data Availability

Data available on reasonable request from the authors.

## Supplementary material

The supplementary material for this article can be found at http://doi.org/10.1017/S0950268825100228.
