# Supplementary material for: Transitioning from microscopy to PCR for protozoa in Norway – Impact on detection of protozoa and helminths: A register study
Source: Epidemiol Infect. 2025 Jun 26;153:e80. doi: 10.1017/S0950268825100228 (PMC12281236; doi:10.1017/S0950268825100228)
Supplement: Sandven et al. supplementary material [file S0950268825100228sup001.docx]

**Transitioning from microscopy to PCR for protozoans in Norway – impact on detection of protozoans and helminths: a register study.**

**Supplementary materials**

Manuscript: Transitioning from microscopy to PCR for protozoans in Norway – impact on detection of protozoans and helminths: a register study.

**Supplementary table S1, PCR kith and detection method**

| **Laboratories/centre** |  | **Nucleic acid extraction** |  | **Detection methods** |  | **Amplification of gene fragments** |
| --- | --- | --- | --- | --- | --- | --- |
| Haukeland  Universitetssjukehus,  Bergen (HUS) |  | MP96 DNA and Viral NA Large volume kit (MagNA Pure 96, Roche) |  | qPCR; FTD Stool Parasites (Launch diagnostics) |  | Unknown |
| St. Olavs University  Hospital, Trondheim  (STO) |  | NucliSENS1®easyMAG1 (BioMerieux) |  | qPCR; Allplex™ GI-Parasite Assay (Seegene) |  | Unknown |
| Universitetssykehuset  Nord Norge, Tromsø  (UNN) |  | STARmag 96x4 Universal Cartridge Kit (Hamilton MICROLAB STARlet) |  | qPCR; Allplex™ GI-Parasite Assay (Seegene) |  | Unknown |
| Nasjonal referanse-  funksjon for molekyl-  ærbiologisk parasitt-  diagnostikk, Ullevål  (OUS |  | QIASymphony DSP  Virus/Pathogen Mini Kit (QIAGEN) |  | qPCR; VIASURE  Cryptosporidium, Giardia & E. histolytica Real-Time Detection Kit (CerTest) |  | 18S rRNA |
| Førde  Sentralsjukehus |  | MP96 DNA and Viral NA Large volume kit (MagNA Pure 96, Roche) |  | qPCR; FTD Stool Parasites (Launch diagnostics) |  | Unknown |

**Supplementary table s2; Episode examined with microscopy (percentage in brackets)**

| Samples/episode |  | prePCR |  | postPCR |
| --- | --- | --- | --- | --- |
| 1 |  | 12916 (89.1) |  | 9633 (77.3) |
| 2 |  | 916 (6.3) |  | 1873 (15.0) |
| 3 |  | 517 (3.6) |  | 675 (5.4) |
| 4+ |  | 145 (1.0) |  | 283 (2.3) |

**Supplementary figure S1. Diagnostic episodes by age cohort, adjusted by proportion of Norwegian population.**

**Supplementary figure S2. Diagnostic episodes by age cohort.**

**
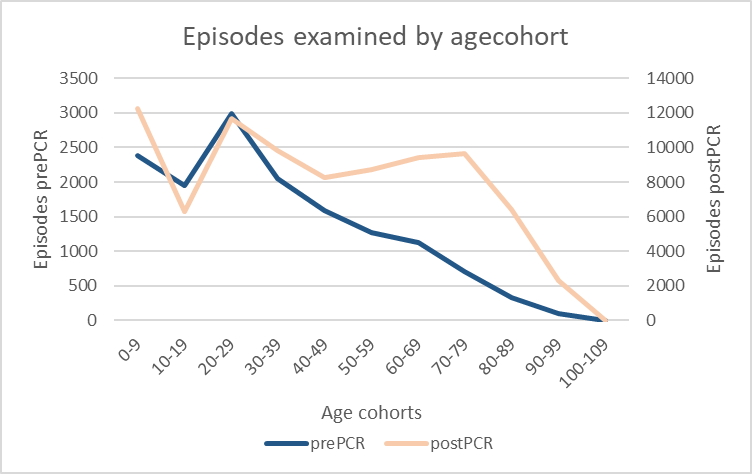
**

**Supplementary table s3; Positive episodes for *Cryptosporidium* pr year by centre**

| Year |  | FSS |  | HUS |  | OUS |  | STO |  | UNN |
| --- | --- | --- | --- | --- | --- | --- | --- | --- | --- | --- |
| 2013 |  |  |  |  |  |  |  |  |  |  |
| 2014 |  |  |  |  |  | 5 |  |  |  |  |
| 2015 |  |  |  |  |  | 13 |  |  |  |  |
| 2016 |  |  |  |  |  | 16 |  | 89 |  |  |
| 2017 |  |  |  | 74 |  | 19 |  | 103 |  |  |
| 2018 |  |  |  | 81 |  | 24 |  | 85 |  |  |
| 2019 |  |  |  | 81 |  | 22 |  | 62 |  |  |
| 2020 |  | 37 |  | 107 |  | 14 |  | 63 |  | 7 |
| 2021 |  | 15 |  | 56 |  | 9 |  | 52 |  | 6 |

**Supplementary table S4. Number of diagnostic episodes examined at least once by microscopy that could detect enteropthogenic helmints**

| **Centre** |  | **prePCR episodes** |  | **postPCR episodes** |  | **prePCR**  **episodes/year** |  | **postPCR**  **episodes/year** |  | **Reduction PrePCR/PostPCR** |  | **Total** |
| --- | --- | --- | --- | --- | --- | --- | --- | --- | --- | --- | --- | --- |
| FSS |  | 1 630 |  | 63 |  | 275 |  | 30 |  | 89 % |  | 1 693 |
| HUS |  | 6 925 |  | 4 936 |  | 2 099 |  | 1 049 |  | 50 % |  | 11 861 |
| OUS |  | 1 060 |  | 4 231 |  | 1 029 |  | 607 |  | 41 % |  | 5 291 |
| STO |  | 1 738 |  | 2 836 |  | 908 |  | 466 |  | 49 % |  | 4 574 |
| UNN |  | 3 141 |  | 398 |  | 512 |  | 213 |  | 58 % |  | 3 539 |
| ***Sum*** |  | ***14 494*** |  | ***12 464*** |  | ***4 823*** |  | ***2 366*** |  | **51%** |  | ***26 958*** |
| *(FSS=Førde Central Hospital, HUS= Haukeland University Hospital, OUS= Oslo University Hospital, STO=Trondheim University Hospital, UNN=University Hospital of North Norway)* | | | | | | | | | | | | |

**Supplementary table S5, Episodes 17 months before covid restrictions in Norway and 17 months after restrictions (positivity rate in percentage).**

|  |  | **17 months pre Covid-19** | | |  | | **17 months with Covid-19** | | | |
| --- | --- | --- | --- | --- | --- | --- | --- | --- | --- | --- |
|  | **Total** | **Micro-scopy** | **PCR** | **Micro +PCR** | |  | | **Micro-scopy** | **PCR** | **Micro +PCR** |
| **Total episodes** | 18286 | 1083 | 15331 | 1873 | |  | | 451 | 13503 | 1071 |
| *Giardia lamblia* | 260 (1.4) | 14 (1.3) | 155 (1.0) | 91 (4.9) | |  | | 6 (1.2) | 84 (0.6) | 37 (3.5) |
| *Cryptosporidium* | 192 (1.0) | 0 (0.0) | 176 (1.1) | 16 (0.8) | |  | | 0 (0.0) | 180 (1.3) | 9 (0.8) |
| *Entamoeba histolytica* | 19 (0.1) | 2 (0.2) | 4 (0.0) | 13 (0.7) | |  | | 1 (0.1) | 0 (0.0) | 4 (0.4) |
| Helminth | 50 (1.7) | 15 (1.4) | NA | 35 (1.9) | |  | | 16 (3.6) | NA | 19 (1.8) |
